# Network Modularity is essential for evolution of cooperation under uncertainty

**DOI:** 10.1038/srep09340

**Published:** 2015-04-07

**Authors:** David A. Gianetto, Babak Heydari

**Affiliations:** 1 School of Systems and Enterprises, Stevens Institute of Technology, Hoboken NJ, USA; 2 Raytheon Space and Airborne Systems, El Segundo CA, USA

## Abstract

Cooperative behavior, which pervades nature, can be significantly enhanced when agents interact in a structured rather than random way; however, the key structural factors that affect cooperation are not well understood. Moreover, the role structure plays with cooperation has largely been studied through observing overall cooperation rather than the underlying components that together shape cooperative behavior. In this paper we address these two problems by first applying evolutionary games to a wide range of networks, where agents play the Prisoner's Dilemma with a three-component stochastic strategy, and then analyzing agent-based simulation results using principal component analysis. With these methods we study the evolution of trust, reciprocity and forgiveness as a function of several structural parameters. This work demonstrates that community structure, represented by network modularity, among all the tested structural parameters, has the most significant impact on the emergence of cooperative behavior, with forgiveness showing the largest sensitivity to community structure. We also show that increased community structure reduces the dispersion of trust and forgiveness, thereby reducing the network-level uncertainties for these two components; graph transitivity and degree also significantly influence the evolutionary dynamics of the population and the diversity of strategies at equilibrium.

The earliest formation of the very building blocks of life, such as RNA, may not have cooperatively formed into complex biological systems without the structural scaffolds required for catalytic processes to evolve^1^,^2^. Just as these building blocks required structured interactions in order to evolve, the structure of social interactions also aide in the emergence of cooperative behavior^3^,^4^,^5^,^6^,^7^.

Models of emergence and survival of cooperation among *selfish* agents are often based on game theoretic social dilemma constructs which capture the interaction of multiple players simultaneously or relatively simple pairwise interaction where each player only interacts with a single player at a time. While the former, usually modeled as a *public goods game*^8^,^9^, is useful for understanding *tragedy of the commons* type situations that challenge prosocial behavior^10^,^11^,^12^, the latter, adopted in this work, allows exploration of a straightforward strategy space and also lends itself directly to network-based simulation methods. Here agents in a pairwise interaction have two actions to choose from, *cooperate* or *defect*. While mutual cooperation provides a Pareto efficient outcome, each player individually gets less than if they were to defect while the other cooperates. This makes mutual defection the Nash Equilibrium of the single shot game, thereby creating a *social dilemma* where both players together receive a higher payoff if they cooperate but maximize their minimum payoff by mutually defecting. The relative payoffs for each combination of player moves can be changed to result in games with different characteristics or dilemma strengths^13^,^14^.

The gloomy prediction of mutual defection made by single-shot social dilemma games contrasts with observations of real world social behavioral in which cooperation between players is common^15^. This difference has been ascribed to repeated interactions and incomplete information^16^, as well as the role of interaction structure as our scope widens from two player interaction to the community level. Spatial and structural effects on behavior have commonly been studied based on evolutionary game theory as the underlying theoretical framework and using agent-based simulation. These studies show that structure has a strong effect on cooperative behavior^17^,^18^,^19^. Local coupling within network structures enables cooperators to cluster together and survive in an environment where defectors have the upper hand^14^; however, defectors are weakened when surrounded by their own kind^20^. If strategy and structure are allowed to evolve together, defection tends to break apart tightly clustered cooperative communities^21^.

Since social ties allow for cooperators to cluster together and improve their survival, are more connections always better? Not necessarily, since many studies have demonstrated the opposite effect. Foundational work by Ref. 22 and Ref. 23 demonstrated that cooperative or *altruistic* genes may dominate a neighborhood when distributed locally due to an increase in the relatedness of individuals; however, this localized dispersion creates competition for resources that naturally limits the spread of these genes. This balance between sufficient structure for interactions between like individuals and the challenges of local competition was later studied by Ref. 17, who found that relatively sparse connectivity encourages cooperation with the intuition that agents with fewer ties value their relations more than agents with more ties and so are more likely to exhibit prosocial behavior in order to maintain those ties. These opposite effects suggest that link distribution rather than merely link density plays a significant role in the survival of cooperation. The effect of link distribution has been shown for some special structures, such as scale-free networks with power-law connectivity, where they support higher cooperation levels than homogeneous networks^24^,^25^,^26^,^27^, though this affect may be limited by participation costs^28^ as well as frequency of interaction where more frequent interaction can negate the cooperative advantage of heterogenous networks over homogeneous^29^. Beyond some of these special structures, the current literature lacks a general understanding of the way link distribution affects cooperation.

Cooperation is not a single-parameter behavior, rather it is composed of a host of components such as trust, reciprocity and forgiveness, that together shape cooperative behavior, yet the majority of studies regard cooperation as a whole. Moreover, many of these works are based on deterministic models that naturally neglect a wide range of mixed strategies that emerge in real-life situations and shape the overall cooperative behavior of a social network. A model suggested by Nowak and Sigmund^30^,^31^ can address both of these issues at the same time by introducing stochastic strategies and breaking them down into three different components of cooperative behavior, (*y*, *p*, *q*), where *y* is the probability of cooperating (*c* in equation (2)) on the first move of a repeated two-player game, *p* is the probability of cooperating when an opponent cooperates, and *q* is the probability of cooperating after an opponent defects (*d* in equation (2)), hence we characterize each of the components of the (*y*, *p*, *q*) triple as *trust*, *reciprocity*, and *forgiveness* respectively for this work^30^. Despite its advantages, to the best of our knowledge, the use of this model for the study of structural effects is limited to Ref. 32 for lattice structures and Ref. 33 who study the effects of cognitive capacity of agents in scale-free networks. In this paper, we use this construct as the building-block of our model and will refer to it as the (*y*, *p*, *q*) model.

The goals of this paper are two-fold. Our first goal is to identify network structural characteristics that have the highest impact on the emergence and survival of cooperative behavior. These characteristics will also give us the necessary tools to tackle the link distribution effect described earlier. Our second goal is to investigate the impact of network structure on individual components of cooperative behavior (namely trust, reciprocity, and forgiveness). To further these goals we employ stochastic agent-based simulations on a wide range of network structures using the (*y*, *p*, *q*) model, and perform multivariate statistical analysis on simulation results. We utilize these methods to provide evidence for the key role of modularity in the evolution of cooperation. Modularity can neutralize high stakes games, similar to how *active linking*, or giving agents the ability to manage their local connections^34^, can reduce the effective strength of cooperative dilemmas. Finally, we show the leadership role of forgiveness in underpinning sustained cooperation in a noisy environment, where forgiveness emerges first in order to dampen the effect of defectors and this allows for reciprocal behavior and trust to emerge.

## Results

Our agent-based^35^ model is built upon an *evolutionary games on graphs*^3^ framework and operates on four levels: pairwise interaction between agents, the execution of an agent's strategy, the evolution of strategy over time, and the network structure through which interactions take place. The lowest level is where decisions are made by agents who receive payoffs in a pairwise game according to equation (4). The *strategy* of each agent, is a three-component stochastic vector, (*y*, *p*, *q*), introduced by Ref. 30. The evolutionary layer is where players imitate the strategies of their neighbors with a probability proportional to their relative payoff (see equation (5)) until an equilibrium of strategies is achieved. Above all, we structure these interactions through a variety of networks where each player occupies a node position and nodes are connected via edges or links through which games are played.

**Figure 1:**
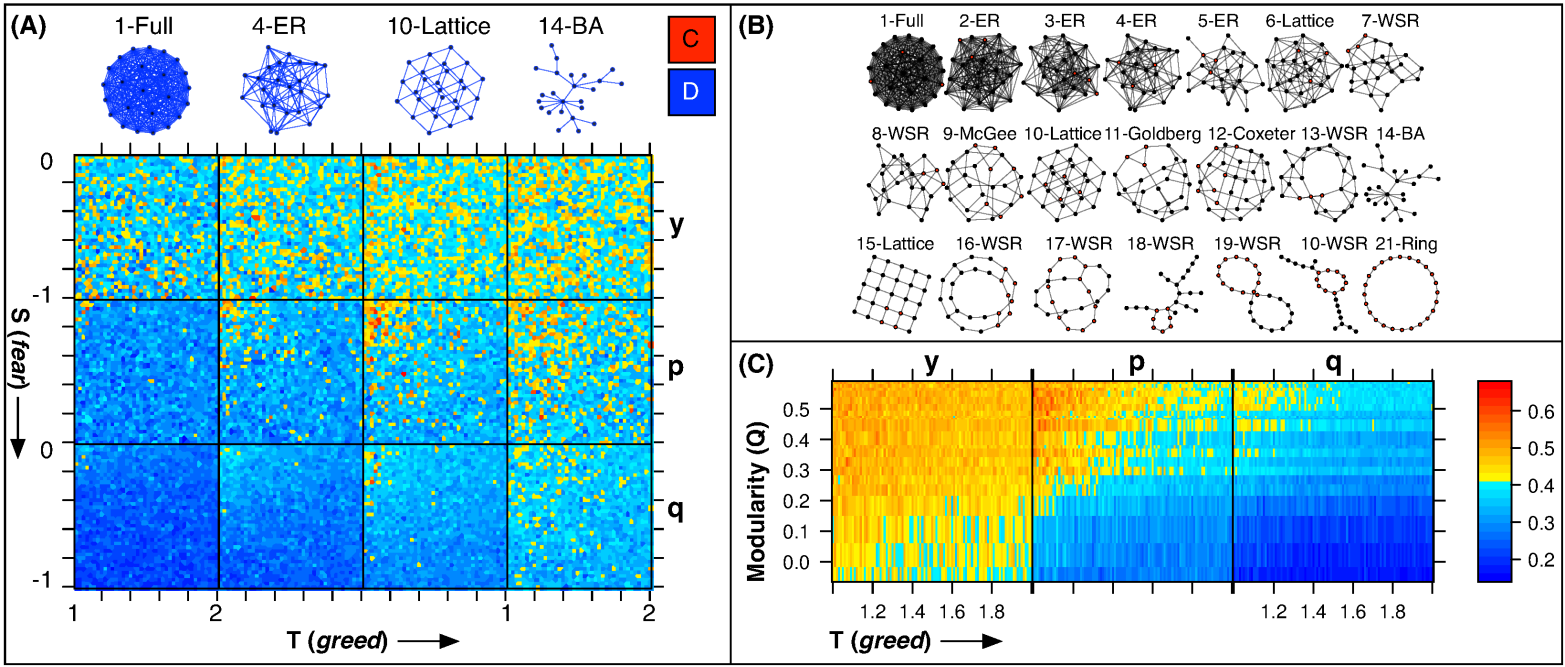
ST-Plane Network Comparison Results. Panel (A) shows a set of 4 × 3 cooperation images of ST-planes covered by equation (3) from a representative 4-structure subset of the 21 structures tested, rows are ordered top to bottom 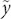, 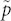, and 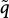 respectively, columns are ordered left to right from the *1-Full* structure to *14-BA*. Each image pixel represents the median cooperation level at coordinates (*T*, *S*) for its respective structure (column) and component (row). Panel (B) is comprised of 21 panels (sorted from low to high average path length), one for each network structure in this work. Panel (C) shows average cooperation of each component for all *S* as a function of modularity (Q) vertically versus temptation to defect *T* horizontally.

The majority of networks we have analyzed have 24 nodes. Our initial sensitivity analysis showed that graphs of approximately this size balance the trade-off between computational efficiency and sensitivity to structural variations needed for this work. Small deviations from this size include lattice structures (10 and 15 in figure 1) which have 27 nodes (3 × 3 × 3) and 25 nodes (5 × 5) respectively, and the Coxeter^36^ graph (12 in figure 1) with 28 nodes. Many of the graphs were generated with a one-dimensional Watts-Strogatz (WSR)^37^ model (7,8,13,16–20 in figure 1), followed by Erdős–Rényi (ER)^38^ (2–5 in figure 1), and lattice (6,10,15 in figure 1). The remainder include a variety of cubic graphs including the McGee graph^39^ (9 in figure 1), a Goldberg *snark*^40^ (*G*_3_ described in^41^ (11 in figure 1)), and the Coxeter^36^ graph (12 in figure 1). The Coxeter and Goldberg graphs are *non-hamiltonian* in that they do not contain a path that visits each vertex exactly once, whereas the McGee graph is hamiltonian^42^. Other tested graphs include a classic ring (21 in figure 1), a fully connected graph (1 in figure 1), and a scale-free graph^43^ (14 in figure 1). The variety of structures we chose was sufficient to determine the key structural characteristics, as borne out by the high variance captured in the multivariate analysis discussed later in this work.

To make our results more reliable and generalizable we relaxed three limiting assumptions that are typically used for reducing computational load and analysis complexity. First, each simulation run draws *new* starting strategies from a random distribution instead of picking from a set of representative strategies, this allows us to explore the strategy space more completely. Second, we randomize all three strategy components so no starting cooperative bias exists. Third, though the simulation system was designed to return only converged results on a run-by-run basis this does not ensure that the final results themselves are stable. Hence we performed a procedure where runs were continually collected until no observable shift in the results was observed. This process required a total of 5.4 core-months of execution time on a Cray XE6 computer to complete successfully.

**Figure 2:**
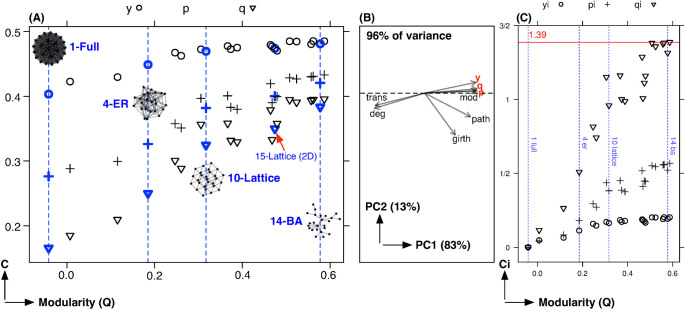
Cooperation Emergence Results Summary. Panel (A) shows the cooperation trend versus modularity for all structures with representative samples shown as dashed vertical lines with each respective structure embedded; the corresponding points for each sample line are shown in blue. The two-dimension square lattice data result is indicated by a red arrow in the figure. Panel (B) is a *biplot*^44^ where vectors (angles shown in table 1) represent the relative correlation of each continuous variant with the first two, most significant, principal components; proportion of variance for each component, is noted on panel (B). A dashed horizontal line on panel (B) is a guide to the eye for comparing the modularity biplot vector with the most significant component (PC1). Panel (C) shows the improvement in each cooperative component (Ci) over the 1-Full graph as modularity increases, where 1 represents a 100% improvement and 0 is the baseline for comparison (the fully connected graph). The red horizontal line is a guide for the eye representing the highest improvement, or 139% for component *p*.

Which are the key network characteristics effecting cooperation emergence? To help answer this question we applied principal component analysis (PCA) of the graph characteristics *degree*, *transitivity*, *girth*, *path length*, and *modularity* in addition to the strategy components 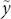, 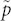, and 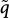 at the final equilibrium of all runs. We then reviewed a *biplot*^44^ representation, shown in figure 2 panel (B), which captures 96% of variation in the data as a whole. We calculated the modularity index based on the measurement of community structure *Q* developed by Ref. 45, where *Q* = 1 is a maximum that indicates a strong community structure, with community detection performed by a random-walk^46^ method.

From table 1, the *modularity* eigenvector correlates well (*cos*(*θ_mod_*) = 0.995) with the first and most significant principal component, due to its alignment with the horizontal axis (*θ_mod_* = 6.75° from table 1); the primary component (PC1) accounts for 83% of variance in the data set as a whole (figure 2 panel (B)). This indicates that modularity is most representative of the primary variation structure in the data (see Ref. 47 for PCA interpretation methods). Moreover, from table 1 the loading, or weight, of modularity is the highest among all network characteristics; degree and transitivity have a similar loading though they are less correlated with PC1 (−0.839 for transitivity and −0.788 for degree). Further, since 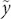, 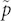, and 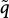 all correlate positively with the modularity, increases in modularity will be met with increases in each of these cooperation components, which is in general agreement with Ref. 48, who study the emergence of total cooperation versus modularity. *Transitivity*, and *degree* are negatively correlated to the cooperation components; however, this holds true even more for trust (*y*), which directly opposes to transitivity (within 5 degrees) and degree. Hence, relatively sparse graphs with less connectivity should encourage more cooperative behavior than more dense highly-connected graphs, this agrees Ohtsuki's *b*/*c* > *k* rule^17^ which Ref. 49 associated with Hamilton's rule^50^ and the notion of *inclusive fitness*^51^ for *bi-transitive* graphs (or graphs that look the same from any pair of nodes). The direction of *girth* also agrees with the analytical model results from Ref. 52 who suggest that as graph girth increases cooperation is enhanced due to the improvement in local cooperator cluster formation; however, since the *girth* vector opposes the *trust* vector in the vertical direction (figure 2 panel (B)) there is a subtle secondary effect present that *reduces* trust relative to reciprocity and forgiveness as girth increases.

From figure 2 panel (A) the positive trend of each individual cooperative element is evident; 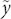 and 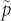 have similar slopes, though 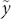 starts at a higher cooperation level. Component 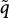 improves at a much higher rate with modularity than the other components, from figure 2, and so begins to mix with 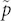 above the 10-Lattice structure. Indeed all components improve with modularity but forgiveness (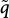) improves by nearly 3/2 (139%) followed by reciprocity (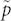) and trust (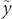) at 1/2 and 1/5 respectively. So as communities form and become more distinct, forgiveness improves the most until a degree of balance is formed with reciprocity.

Cooperation differences across the PD game ST-plane (figure 6) are largely non-existent for 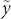 (top row of figure 1 panel (A) and left column of panel (C)); however, 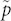 shows significant improvement in the low stakes region (upper left corner of ST-plane). This effect is somewhat evident in the 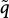 (bottom row of figure 1 panel (A)) as well. By averaging across all *fear* (*S*) the effect of modularity on each component is more clear, shown in figure 1 panel (C). Though the highest cooperation levels are attained in 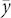 and 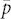, the cooperation gradient is highest for 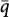 where the games with the lowest stakes stand to improve the most with modularity.

**Figure 3:**
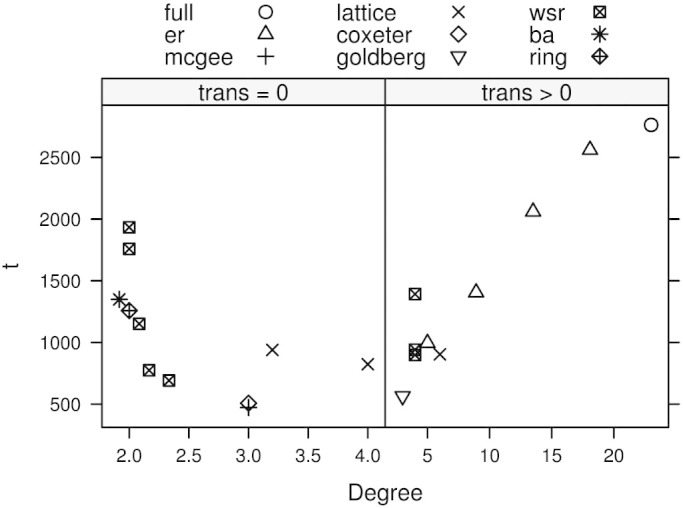
Run Convergence. The figure shows the effect of *degree* on convergence time 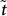 conditioned on transitivity, where the left panel has a transitivity of zero and the right panel has a transitivity >0.

There are significant differences in the evolutionary dynamics of the different structures tested. Indeed, run convergence time varies widely across different networks and its structural dependency can provide some interesting insights. From figure 3, for graphs with zero transitivity, higher average degree graphs tend to converge quicker than lower degree graphs; however, once the graphs become more transitive the convergence time builds significantly–to a factor of five over the fastest converging structure. Since a more transitive graph is more locally homogenous, that is the local structure is less distinctive across the network from a given local agent's perspective^49^, it follows that this lack of distinction requires more time to converge since similar strategies may become locally stable across the network but will compete in the margins for global convergence.

## Discussion

All strategic components show improvement with network modularity; however, *forgiveness* shows the most sensitivity to structure which results in a factor of 2.4 improvement in the probability that an agent forgives 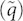 a defection action by an opponent for the 14-BA graph (*Q* = 0.6) compared to an unstructured (well-mixed) population (*Q* = 0). This makes intuitive sense because forgiveness is necessary in order for cooperation to evolve since the initial conditions are equally populated by defectors and cooperators. Without forgiveness there would be no cooperative acts to reciprocate and so higher levels of forgiveness improvement relative to reciprocity and trust indicates that forgiveness underpins the other cooperative behaviors. Moreover, forgiveness can more easily evolve in more modular structures since community boundaries and limited interaction protect the more forgiving strategies from being exploited by agents in other communities. This finding naturally extends the previous literature on the role of *forgiveness* in the emergence of cooperation, first demonstrated by Ref. 31 for an unstructured population and later shown to be even more significant by Ref. 32 who showed a factor of 2 improvement in forgiveness for a variety of square lattice structures compared to the unstructured model. Our results agree with this factor of 2 improvement; however, we extend this result to show that the probability of forgiveness can be even higher for structures with higher levels of modularity than lattice structures.

The impact of motivation, or payoffs, changes with modularity as well. Indeed, increasing community structure also increases trust behaviors across a range of fearful and greedy motivators. In other words, community structure improves societal trust in-general, because of the invariance of the improvement in 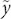 to *S* and *T* as modularity increases, shown in figure 1. Since the strategies are *reactive* in that they react to past moves of their opponents, initial distrusting moves cause an enduring effect that reduces cooperative levels. With higher degree networks that are less modular the effect of these distrusting moves is felt globally across the network; however, with modular structure the impact is contained to a local community level. The level of motivation, or the *stakes* of the game have little effect on trust because the decision to trust, as we have modeled it, must be made with no information as to the benefits and risks of trusting and so this result is consistent with emergent *unconditional* trusting behavior.

**Figure 4:**
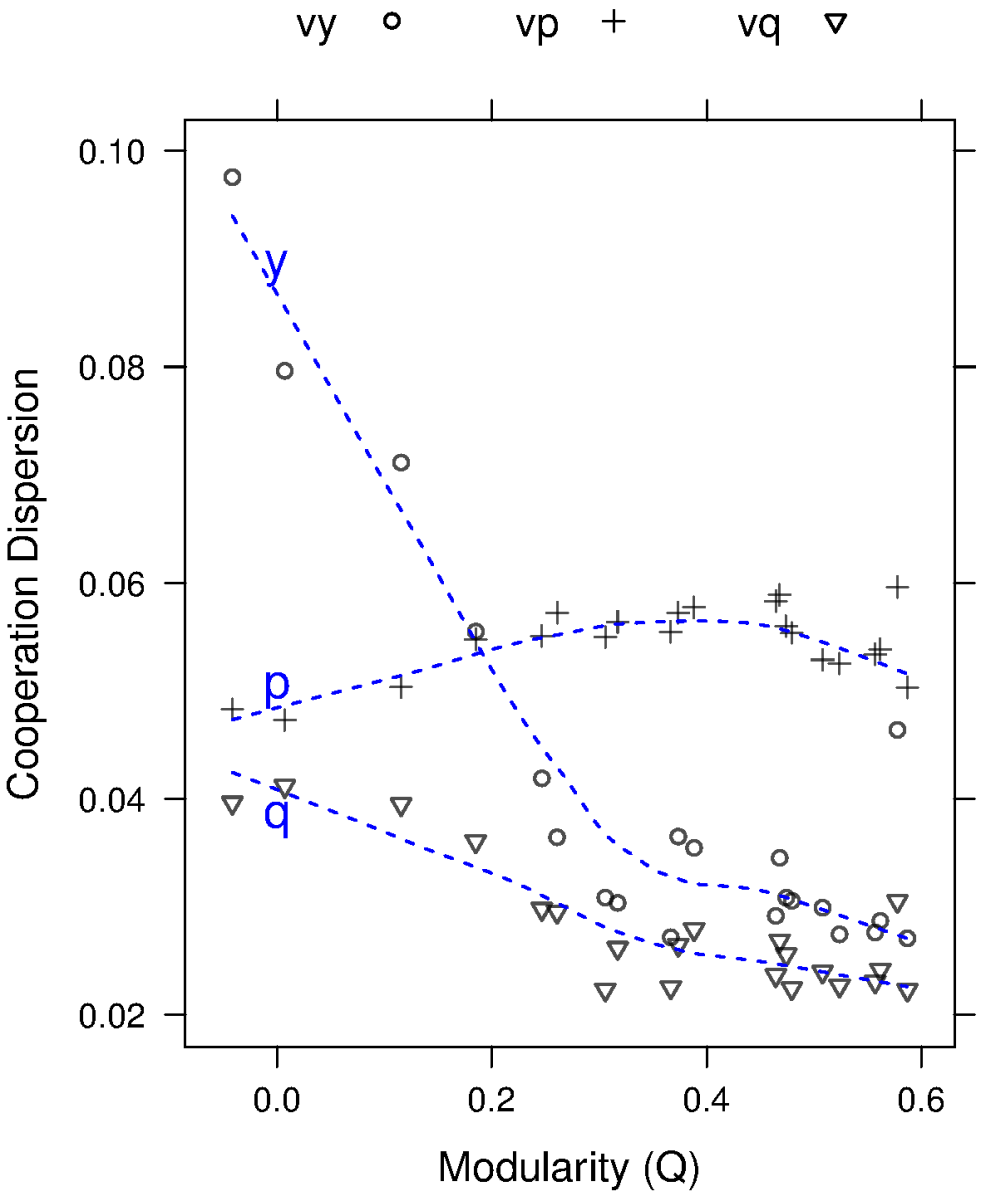
Cooperation Dispersion. The figure shows the dispersion (*σ*/*μ*) of each cooperation component versus modularity. The dashed blue lines trace a local fit for each component.

The cooperation gradient across the ST-plane in figure 1 panel (A) is consistent with that of Refs. 27, 53,27, 53; however, the gradient dissolution with modularity is a novel effect. The differences in the flattening of the gradient, or the reduction in *dispersion* (*σ*/*μ*) across the ST-plane as a function of modularity is shown in figure 4. Here the dispersion in reciprocity 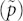 is nearly unaffected by modularity, however trust 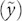 dispersion reduces dramatically along with forgiveness 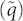. This suggests that trust and forgiveness behaviors are moderated by modularity or modularity reduces the uncertainty of system-wide global trust and forgiveness behaviors under uncertain conditions.

**Figure 5:**
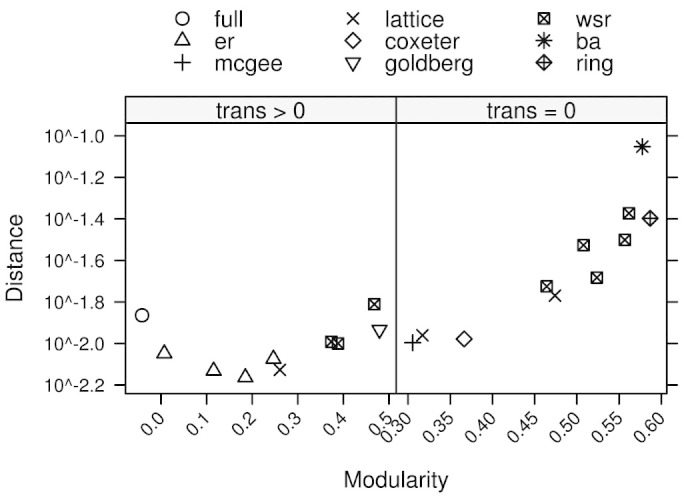
Strategy Diversity. The figure shows the median euclidian distance between strategies at equilibrium as a function of modularity, conditioned on transitivity, where the left panel has a transitivity of >0 and the right panel has a transitivity of zero. The vertical axis is scaled logarithmically.

Why does the influence of payoffs on behaviors reduce as modularity increases? Community structures, or modularity, allow more diverse strategies to evolve and become stable since community boundaries reduce the influence of outside strategies on conditional cooperators. Conversely modularity can be thought of as an emergent property that improves survival in the presence of diverse environmental effects and reactive agents^54^,^55^. We summarize the effect of these diverse strategies as a flattening of the ST-plane gradient. The significant increase in strategy diversity is shown in figure 5 where transitive graphs tend to have less diverse strategies than non-transitive graphs. Intuitively this is because the similar local structures of the transitive graphs provide fewer local niches that support the evolution of diverse strategies.

We summarize effect of modularity and incentives (payoff) together in figure 1 panel (C). Here, the angular gradient can be interpreted with respect to modularity or payoff. From a modularity perspective, less modularity is needed to attain the same level of cooperation improvement when stakes are relatively low; however, as stakes increase more modularity is needed to sustain the same cooperative behaviors. From a payoff perspective, the influence of payoffs on sustained cooperative behaviors decreases as modularity increases, or modularity has the ability to neutralize the effect of payoffs on behaviors such that relatively high levels of cooperation can be sustained into even the strongest social dilemmas (high *T*).

The effect of modularity on cooperative behavior can also be shown analytically, using a simplified model (see *Methods*). In this deterministic model, we assume that the temptation to defect is equal to *b* and the higher this temptation the more modular a connected component needs to be in order for cooperation to survive and propagate. Defining *α* as the ratio of internal community links *k_in_* and external links *k_out_* for node *i*, we can show that
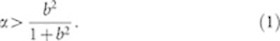


Equation (1) aligns qualitatively with the modularity gradient in figure 1 panel (C), where increased modularity reduces the effect of higher *T*(*b*) on cooperation for *p* and *q*.

**Figure 6:**
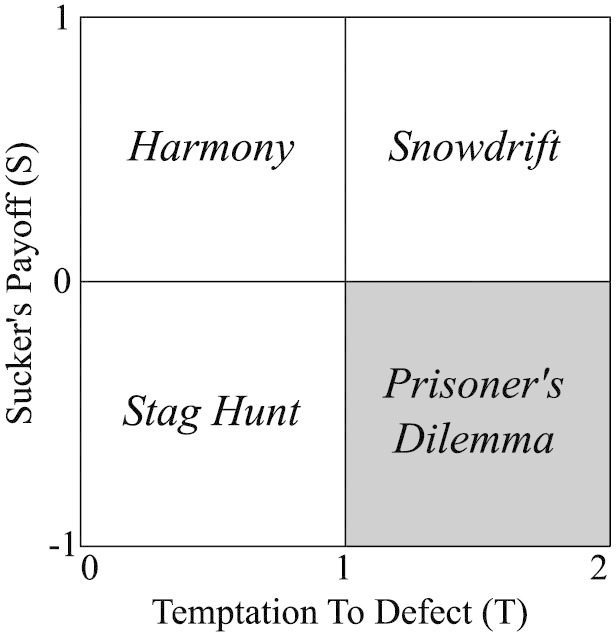
Stylized Games Payoff Structure. The figure, adapted from Ref. 60, presents a two-dimensional plane of payoffs where *S* is received by by a player (or paid from a player when *S* < 0) for cooperating while an opponent defects and *T* is received by a player who defects while an opponent cooperates. Hence, *S* is representative of fear motivation (the more negative the more fear) and *T* is representative of greedy motivation (the more positive the more greed)^33^. The shaded region represents the payoff range of the current work, namely the *Prisoner's Dilemma* game.

Additional insight can be gained by expanding the present work to other two-player games such as the Snowdrift and Stag Hunt games and studying the effect of modularity across the wider multi-game *greed-fear* (*T* − *S*) plane of payoffs, shown in figure 6. Initial conditions may also be varied including studying the the changes in structural effects when initially *mean* or *nice* strategies are present. Does the central role of modularity hold for a wider variety of strategies? The three-component strategy framework can be extended to four, from Ref. 31, where (*p*1, *p*2, *p*3, *p*4) are the probabilities to cooperate after a (*C*, *C*), (*C*, *D*), (*D*, *C*), and (*D*, *D*) respectively in the prior round as well as the probability to cooperate in the first round, giving a five component strategy that adds the notion of self-knowledge relative to an opponent's past moves.

Finally, given that selection strength can alter the dynamics of evolving structured populations^56^ future work should also focus on testing the robustness of the present results with both strong and weak selection through the use of the more common Fermi evolutionary rule.

## Methods

Our model operates at four levels: game (behavioral), strategy, evolution, and network. At the behavioral level we first adopt a simplified symmetric two-player repeated game, shown in equation (2) described by Ref. 57, 58, where the defecting player receives *T* (temptation to defect) if their opponent cooperates (*c*) but receives *P* = 0 if their opponent defects (*d*), which is the punishment for mutual defection. In this work we limit the scope of our analysis to the Prisoner's Dilemma range of payoffs (lower right quadrant of figure 6) and reserve the other game structures for future work.

If both players mutually cooperate they each receive *R* = 1; however, the cooperating player receives *S* < *R* (suckers payoff) if their opponent defects. Though *P* and *R* are fixed, we vary *S* and *T* over a range of payoffs within the *Prisoner's Dilemma* (PD) game, which is characterized by 1 < *T* < 2 and −1 < *S* < 0^3^, shown in figure 6.
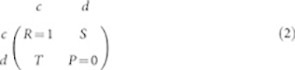


In our simulations we draw *T* and *S* from uniform distributions within the range of PD payoffs, shown in equation (3).
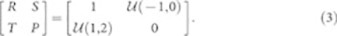


All scores that players receive in repeated games are not counted equally; each iteration *i* of the game contributes less than the previous iteration to the total score since it is common to discount the future relative to the present^14^. We apply a discount factor *w* to account for this such that the *i*'th round is worth a mere *w^i^* of the first round score. The player with the highest total score upon completion is the winner. Equation (4) describes how the payoff Π*_o_* is determined, where *k_o_* is the degree of node *o*, *w_o_* is the node's discount factor which is drawn from a uniform distribution 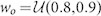, *g* is the number of repeated games played (fixed at 50), and 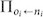 is the payoff received by player *o* versus its neighbor *n* in game *i*.
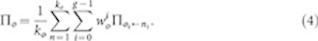


For the logic behind player's behaviors or their *strategy* we apply the (*y*, *p*, *q*) cooperation component framework from Ref. 30. Past studies usually simplify this framework by assuming that the first component *y* is fixed at *y* = 1 (i.e., always cooperate on the first move), either with the rationale that a single move becomes less important in an infinitely repeated game^31^ or that a two-strategy framework lends itself well to a two dimensional representation^59^. Since our purpose is to study how structure impacts trust as well as reciprocity and forgiveness we do not assume *y* is fixed and so begin each simulation with a *completely random* (*y*, *p*, *q*) *strategy variant* for each network node. This leads both to unique insights and to increased generality of our results since there is no starting bias, whereas the assumption of *y* = 1 is a certainly a cooperative one. Moreover, our strategy space is highly stochastic since the initial *y*, *p*, *q* values are each drawn from a uniform distribution for every node, resulting in millions of starting strategies across the simulation runs performed for this work. This also effectively eliminates distribution effects between cooperators and defectors, described by Ref. 53.

The evolutionary process is as follows. We first initialize each node in the network to an initial strategy *s_o_* which is defined by the triple (*y_o_*, *p_o_*, *q_o_*) each component of which is drawn from a uniform distribution 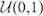. From then on, for each time step, the population is evolved through a pairwise comparison rule where the game score of each node in the network Π*_o_* is sequentially compared with a randomly chosen neighbor Π*_n_*; strategies are then updated with the probability *W*(*s_n_* → *s_o_*) defined in equation (5), which was introduced by Ref. 24.
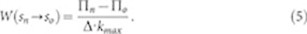


The *k_max_* variable in equation (5) is the maximum degree of nodes *o* and *n*, and Δ = *T* − *S* for the PD game. Equation (5) has the nice property of evolving both high and low degree nodes at the same rate, which the commonly-used *Fermi* function lacks^6^. This is an important characteristic for this work because of the variety of network structures we test; however, equation (5) gives up the ability to adjust the evolutionary selection strength *β*, present in the *Fermi* function but we keep evolutionary strength constant for this work and so are unaffected by the inability to control it.

Using equation (5), the evolutionary process continues for multiple generations until no more than *one* node updates its strategy within a sequential time window of 100 generations. To aide in convergence we also provide a strategy adoption fixed noise threshold 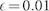, below which nodes will not update their strategy.

Finally, nodes that switch strategies adopt the average of their present strategy and the chosen new strategy. This method greatly enhances the simulation convergence time (>10× faster over a simple copy process) by reducing the cycling of strategies through the network while also exploring a greater variety of strategies.

### Derivation of modularity relation

To help understand the effect of modularity on agents as game stakes increase we have derived the relation equation (1) which shows that the more temptation to defect (*b*), the more modular (*α*) the component needs to be in order for cooperation to survive and propagate, which aligns qualitatively with the modularity gradient in figure 1 panel (C). This section details the derivation of this relation.

Our goal is to demonstrate that modularity increases the chance of survival and expansion of cooperative behavior in a network. We use a Prisoner's Dilemma framework with *R* = 1 and *T* = *b* > 1 and assume there are only two deterministic payoff structures: Cooperation (*S_i_* = 1) and Defection (*S_i_* = 0). The agents learn from their more successful agents with a probability proportional to the difference in their aggregate payoffs. We test the structural conditions under which a group of cooperators cannot be *invaded* by defectors but a group defectors have a finite probability of becoming cooperators.

For the first goal, we pick any connected component of cooperators within the network and show that this connected component is a *module*. For each node in this component, either all the neighbors are already cooperators, meaning that the node is fully embedded in the component, or the node has a mixed set of neighbors, in which case we call it a *boundary* node. Since the *boundary* node is a member of the connected component of cooperators, it cannot have any cooperative neighbor that is outside of the component. If the degree of the boundary node *i* is *k_i_*, the neighborhood can be divided into a set of cooperating neighbors *k_in_* and defecting neighbors *k_out_*.

In order for the component to stay cooperative, every boundary node *i* needs to have a larger aggregate payoff than any of its defecting neighbors (node *j*). The payoff for node *i* is simply equal to *k_in_* and the payoff for node *j* is 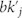. We need to have 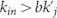. The variable 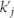, the number of cooperating agents of node *j*, can take any number between 1 (since *i* already is a cooperator) to *k_j_*, the degree of node *j*; this condition must hold especially for the 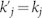 since it is the worst case for cooperator survival. Thus we have:
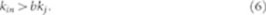


Now consider the same component and assume that all the members are defectors. We want to identify the structural condition under which boundary nodes, of this *defector* component, have a finite chance of becoming cooperators. These boundary nodes exclusively derive their payoffs from cooperators and all of them lie *outside* of the defector component. This score is *bk_out_* and needs to be smaller than the score of a cooperative neighbor. The latter is simply *k*″*_j_*. So for node *i* we have:
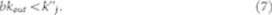


We also have 0 ≤ *k*″*_j_* ≤ *k_j_* − 1. Thus we have *bk_out_* < *k_j_* − 1. Combining this with equation (6) gives:
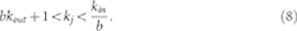


This gives a condition for the relative value of *k_in_* and *k_out_* for node *i*. Assuming that *bk_out_* is much larger than 1, and defining *α* as the ratio of *k_in_* to *k_i_*, that is a measure of how modular the connected component is, equation (8) turns into:
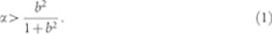


## Author Contributions

Author D.G. created all figures and wrote the main manuscript text before the *Derivation of Modularity Relation* section, which was written by author B.H. Author D.G. created the model, ran simulations and analyzed results, the work was supervised by B.H. who also helped refine the research questions.

## Figures and Tables

**Table 1:** Correlation of Network Parameters to Modularity

parameter	PC1 loading	*θ*	*cos*(*θ_mod_* − *θ_param_*)
transitivity	−0.360	−147	−0.899
degree	−0.366	−142	−0.856
girth	0.224	−74.1	0.159
path length	0.335	−53.0	0.504
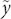	0.375	28.3	0.930
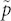	0.384	11.7	0.996
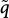	0.384	5.71	1.000
modularity	**0.373**	6.75	1.000

The PC1 Loading column shows the relative contribution weight of each parameter to PC1 (highest independent variable loading is in bold), which is a linear combination of each parameter and weight. The angle of each parameter eigenvector (from figure 2 panel (B)) is shown in column *θ* (degrees). The correlation between each parameter in the biplot by the cosine of the angle difference between the modularity eigenvector and each respective parameter eigenvector, or *cos*(*θ_mod_* − *θ_param_*).

## References

[b1] Vaidya N. *et al.* Spontaneous network formation among cooperative rna replicators. Nature 491, 72–77 (2012). 23075853 10.1038/nature11549

[b2] Attwater J. & Holliger P. Origins of life: The cooperative gene. Nature 491, 48–49 (2012). 23075847 10.1038/nature11635

[b3] Szabó G. & Fáth G. Evolutionary games on graphs. Phys. Rep. 446, 97–216 (2007).

[b4] Santos F. C., Rodrigues J. F. & Pacheco J. M. Graph topology plays a determinant role in the evolution of cooperation. Proc. Biol. Sci. 273, 51–55 (2006). 16519234 10.1098/rspb.2005.3272 PMC1560002

[b5] Perc M. & Grigolini P. Collective behavior and evolutionary games an introduction. Chaos Solitons Fractals 56, 1–5 (2013).

[b6] Perc M. & Szolnoki A. Coevolutionary games-a mini review. BioSystems 99, 109–125 (2010). 19837129 10.1016/j.biosystems.2009.10.003

[b7] Castellano C., Fortunato S. & Loreto V. Statistical physics of social dynamics. Rev. Mod. Phys. 81, 591–646 (2009).

[b8] Gomez-Gardeñes J., Romance M., Criado R., Vilone D. & Sánchez A. Evolutionary games defined at the network mesoscale: the public goods game. Chaos 21, 016113; 10.1063/1.3535579 (2011). 21456855

[b9] Peña J. Group-size diversity in public goods games. Evolution 66, 623–636 (2012). 22380428 10.1111/j.1558-5646.2011.01504.x

[b10] Lejano R. P. & Fernandez de Castro F. Norm, network, and commons: The invisible hand of community. Environ. Sci. Policy 36, 73–85 (2014).

[b11] Perc M., Gómez-Gardeñes J., Szolnoki A., Floría L. M. & Moreno Y. Evolutionary dynamics of group interactions on structured populations: a review. J. R. Soc. Interface 10, 20120997; 10.1098/rsif.2012.0997 (2013). 23303223 PMC3565747

[b12] Archetti M. & Scheuring I. Review: Game theory of public goods in one-shot social dilemmas without assortment. J. Theor. Biol. 299, 9–20 (2012). 21723299 10.1016/j.jtbi.2011.06.018

[b13] Von Neumann J. & Morgenstern O. Theory of games and economic behavior. Bull. Amer. Math. Soc 51, 498–504 (1945).

[b14] Axelrod R. & Hamilton W. D. The evolution of cooperation. Science 211, 1390–1396 (1981). 7466396 10.1126/science.7466396

[b15] Ostrom E. Collective action and the evolution of social norms. J. Econ. Perspect. 14, 137–158 (2000).

[b16] Kreps D. M., Milgrom P., Roberts J. & Wilson R. Rational cooperation in the finitely repeated prisoners' dilemma. JET 27, 245–252 (1982).

[b17] Ohtsuki H., Hauert C., Lieberman E. & Nowak M. A. A simple rule for the evolution of cooperation on graphs and social networks. Nature 441, 502–505 (2006). 16724065 10.1038/nature04605 PMC2430087

[b18] Kearns M., Suri S. & Montfort N. An experimental study of the coloring problem on human subject networks. Science 313, 824–827 (2006). 16902134 10.1126/science.1127207

[b19] Hanaki N., Peterhansl A., Dodds P. S. S. & Watts D. J. J. Cooperation in evolving social networks. Manag. Sci. 53, 1036–1050 (2007).

[b20] Szolnoki A. & Perc M. Defection and extortion as unexpected catalysts of unconditional cooperation in structured populations. Sci. Rep. 4, 5496; 10.1038/srep05496 (2014). 24975112 PMC4074784

[b21] Cavaliere M., Sedwards S., Tarnita C. E., Nowak M. A. & Csikász-Nagy A. Prosperity is associated with instability in dynamical networks. J. Theor. Biol. 299, 126–38 (2012). 21983567 10.1016/j.jtbi.2011.09.005 PMC3298632

[b22] Wilson D. S., Pollock G. B. & Dugatkin L. A. Can altruism evolve in purely viscous populations? Evol. Ecol. 6, 331–341 (1992).

[b23] Taylor P. D. Altruism in viscous populations an inclusive fitness model. Evol. Ecol. 6, 352–356 (1992).

[b24] Santos F. C. & Pacheco J. Scale-free networks provide a unifying framework for the emergence of cooperation. PRL 95, 098104; 10.1103/PhysRevLett.95.098104 (2005). 16197256

[b25] Gómez-Gardeñes J., Campillo M., Floría L. & Moreno Y. Dynamical organization of cooperation in complex topologies. PRL 98, 108103; 10.1103/PhysRevLett.98.108103 (2007). 17358570

[b26] Santos F. C. & Pacheco J. M. A new route to the evolution of cooperation. J. Evol. Biol. 19, 726–33 (2006). 16674569 10.1111/j.1420-9101.2005.01063.x

[b27] Santos F. C., Pinheiro F. L., Lenaerts T. & Pacheco J. M. The role of diversity in the evolution of cooperation. J. Theor. Biol. 299, 88–96 (2012). 21930134 10.1016/j.jtbi.2011.09.003

[b28] Masuda N. Participation costs dismiss the advantage of heterogeneous networks in evolution of cooperation. Proc. Biol. Sci. 274, 1815–21 (2007). 17504741 10.1098/rspb.2007.0294 PMC2270926

[b29] Maciejewski W., Fu F. & Hauert C. Evolutionary game dynamics in populations with heterogenous structures. PLoS Comput. Biol. 10, e1003567; 10.1371/journal.pcbi.1003567 (2014). 24762474 PMC3998889

[b30] Nowak M. & Sigmund K. The evolution of stochastic strategies in the prisoner's dilemma. Acta Appl. Math. 20, 247–265 (1990).

[b31] Nowak M. A. & Sigmund K. Tit for tat in heterogeneous populations. Nature 355, 250–253 (1992).

[b32] Grim P. The greater generosity of the spatialized prisoners dilemma. J. Theor. Biol. 173, 353–359 (1995).

[b33] Vukov J., Santos F. C. & Pacheco J. M. Cognitive strategies take advantage of the cooperative potential of heterogeneous networks. New J. Phys. 14, 063031; 10.1088/1367-2630/14/6/063031 (2012).

[b34] Pacheco J., Traulsen A. & Nowak M. Coevolution of strategy and structure in complex networks with dynamical linking. PRL 97, 258103; 10.1103/PhysRevLett.97.258103 (2006). PMC2430061 17280398

[b35] Axelrod R. Simulation in the social sciences. Handbook of Research on Nature-Inspired Computing for Economics. and Management. , Rennard J.-P., ed. (ed.), 90–100; 10.4018/978-1-59140-984-7.ch007 (IGI Global, HersheyPA, 2007).

[b36] Coxeter H. S. M. My graph. Proc. London Math. Soc. 3, 117–136 (1983).

[b37] Watts D. & Strogatz S. Collective dynamics of small-world networks. Nature 393, 440–442 (1998). 9623998 10.1038/30918

[b38] Erdos P. & Rényi A. On the evolution of random graphs. Magyar Tud. Akad. Mat. Kutató Int. Közl 5, 17–61 (1960).

[b39] McGee J. W. & Rodger C. A. Embedding coverings of 2-paths with 3-paths. Discrete Math. 284, 217–223 (2004).

[b40] Steffen E. Classifications and characterizations of snarks. Discrete Math. 188, 183–203 (1998).

[b41] Ghebleh M. The circular chromatic index of goldberg snarks. Discrete Math. 307, 3220–3225 (2007).

[b42] Angluin D. & Valiant L. G. Fast probabilistic algorithms for hamiltonian circuits and matchings. In: Proceedings of the ninth annual ACM symposium on Theory of computing 30–41; 10.1145/800105.803393 (ACM, 1977).

[b43] Albert R. & Barabási A. L. Statistical mechanics of complex networks. Rev. Mod. Phys. 74, 47–97 (2002).

[b44] Gabriel K. R. The biplot graphic display of matrices with application to principal component analysis. Biometrika 58, 453–467 (1971).

[b45] Newman M. E. J. & Girvan M. Finding and evaluating community structure in networks. Phys Rev E Stat Nonlin Soft Matter Phys 69, 026113; 10.1103/PhysRevE.69.026113 (2004). 14995526

[b46] Pons P. & Latapy M. Computing communities in large networks using random walks. J. Graph Algorithms Appl. 10, 191–218 (2006).

[b47] Rencher A. C. & Christensen W. F. Principal Component Analysis. Methods of multivariate analysis 2nd edn., vol. 709, 405–433; 10.1002/0471271357 (John Wiley & Sons, 2012).

[b48] Marcoux M. & Lusseau D. Network modularity promotes cooperation. J. Theor. Biol. 324, 103–108 (2013). 23261393 10.1016/j.jtbi.2012.12.012

[b49] Taylor P. D., Day T. & Wild G. Evolution of cooperation in a finite homogeneous graph. Nature 447, 469–472 (2007). 17522682 10.1038/nature05784

[b50] Hamilton W. D. The genetical evolution of social behaviour. i. J. Theor. Biol. 7, 1–16 (1964). 5875341 10.1016/0022-5193(64)90038-4

[b51] Taylor P. D. & Maciejewski W. Hamilton's inclusive fitness in finite-structured populations. Phil. Trans. R. Soc. B 369, 20130360; 10.1098/rstb.2013.0360 (2014). 24686932 PMC3982662

[b52] Manshadi V. H. & Saberi A. Dynamics of prisoner's dilemma and the evolution of cooperation on networks. In: Proceedings of the 3rd Innovations in Theoretical Computer Science Conferences 227–235; 10.1145/2090236.2090256 (ACM, 2012).

[b53] Roca C. P., Cuesta J. A. & Sánchez A. Effect of spatial structure on the evolution of cooperation. Phys Rev E Stat Nonlin Soft Matter Phys 80, 046106; 10.1103/PhysRevE.80.046106 (2009). 19905389

[b54] Heydari B. & Dalili K. (in press) Emergence of modularity in system of systems: Complex networks in heterogeneous environments. IEEE Syst. J. 1–9; 10.1109/jsyst.2013.2281694 (2013).

[b55] Gianetto D. A. & Heydari B. (in press) Catalysts of cooperation in system of systems: The role of diversity and network structure. IEEE Syst. J. 1–9; 10.1109/jsyst.2013.2284959 (2013).

[b56] Segbroeck S. V., Santos F. C., Lenaerts T. & Pacheco J. M. Selection pressure transforms the nature of social dilemmas in adaptive networks. New J. Phys. 13, 013007; 10.1088/1367-2630/13/1/013007 (2011).

[b57] Nowak M. A. & May R. M. Evolutionary games and spatial chaos. Nature 359, 826–829 (1992).

[b58] Nowak M. A. Evolving cooperation. J. Theor. Biol. 299, 1–8 (2012). 22281519 10.1016/j.jtbi.2012.01.014

[b59] Fischer I. The emergence of reactive strategies in simulated heterogeneous populations. Theory Decis. 55, 289–314 (2003).

[b60] Roca C. P., Cuesta J. A. & Sánchez A. Evolutionary game theory: Temporal and spatial effects beyond replicator dynamics. Phys. Life Rev. 6, 208–49 (2009). 20416850 10.1016/j.plrev.2009.08.001

